# Molecular dynamics simulations indicate an induced-fit mechanism for LSD1/CoREST-H3-histone molecular recognition

**DOI:** 10.1186/2046-1682-6-15

**Published:** 2013-11-25

**Authors:** Nadeem A Vellore, Riccardo Baron

**Affiliations:** 1 Department of Medicinal Chemistry, College of Pharmacy, and The Henry Eyring Center for Theoretical Chemistry, The University of Utah, Salt Lake City, UT 84112-5820, USA

**Keywords:** Epigenetics, Chromatin remodeling, Computer simulation, Conformational clustering, Conformational ensemble, Histone, Protein binding, Statistical test, Kolmogorov-Smirnov statistics

## Abstract

**Background:**

Lysine Specific Demethylase (LSD1 or KDM1A) in complex with its co-repressor protein CoREST catalyzes the demethylation of the H3 histone N-terminal tail and is currently one of the most promising epigenetic targets for drug discovery against cancer and neurodegenerative diseases. Models of non-covalent binding, such as lock and key, induced-fit, and conformational selection could help explaining the molecular mechanism of LSD1/CoREST-H3-histone association, thus guiding drug discovery and design efforts. Here, we quantify the extent to which LSD1/CoREST substrate binding is consistent with these hypothetical models using LSD1/CoREST conformational ensembles obtained through extensive explicit solvent molecular dynamics (MD) simulations.

**Results:**

We find that an induced-fit model is the most representative of LSD1/CoREST-H3-histone non-covalent binding and accounts for the local conformational changes occurring in the H3-histone binding site. We also show that conformational selection – despite in principle not ruled out by this finding – is minimal, and only relevant when global properties are considered, e.g. the nanoscale motion of the LSD1/CoREST clamp.

**Conclusion:**

The induced-fit mechanism revealed by our MD simulation study will aid the inclusion of protein dynamics for the discovery and design of LSD1 inhibitors targeting the H3-histone binding region. On a general basis, our study indicates the importance of using multiple metrics or selection schemes when testing alternative hypothetical mechanistic models of non-covalent binding.

## Background

**Figure 1:**
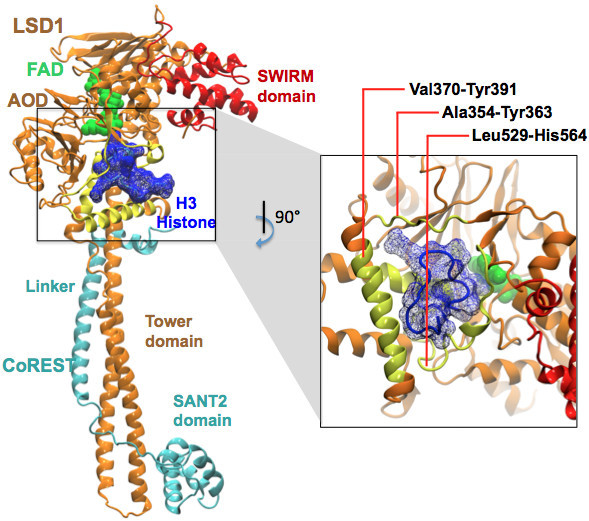
**Structural biology of LSD1/CoREST complex.** The crystal structure of LSD1/CoREST complex bound to the H3-histone N-terminal tail (PDB entry 2V1D). LSD1 (orange) consists of the amine oxidase (AO) domain, SWIRM (red), and Tower domains. CoREST (cyan) consists of the linker and SANT2 domains. The figure highlights the first sixteen N-terminal residues of the H3-histone substrate (blue mesh surface), the H3-tail binding region (yellow) and the FAD cofactor (green spheres).

Lysine Specific Demethylase-1 (LSD1) is an epigenetic target of outstanding interest for the discovery of drugs against cancer [1-6] and neurodegenerative disorders [7]. LSD1 associates with its co-repressor protein (CoREST) and demethylates the mono- or di methylated Lys4 residue on the H3-histone N-terminal tail using a flavin adenosine dinucleotide (FAD) cofactor [3,8,9]. Figure 1 summarizes the structural organization of the human LSD1/CoREST complex bound to the N-terminal tail of the H3-histone protein [10]. However, little knowledge is currently available on the atomistic details of the dynamic binding mechanism employed in LSD1-chromatin recognition, thus hampering the development of novel inhibitors and molecular probes targeting this process for pharmacological goals.

Using extensive LSD1/CoREST conformational ensembles generated by explicit solvent molecular dynamics (MD) simulation [11], we have previously shown that LSD1/CoREST is a highly dynamic nanoscale clamp with opening and closing amplitudes on the nanometer scale. Our previous studies indicated that the H3-histone N-terminal tail peptide binding to LSD1 acts as an allosteric modulator by reducing the rotation of the amine oxidase (AO) domain with respect to the Tower domain [11].

Various molecular recognition models help the interpretation of possible mechanisms of receptor-ligand binding, thus far not applied in the context of LSD1/CoREST recognition of binding partners. In 1894, a first *lock-and-key* model was proposed by Fischer to characterize non-covalent receptor-binding based on the shape complementarity of ligand molecules with the binding site of a rigid receptor [12]. Soon after, frequent observations emerged demonstrating that high binding affinities need not be correlated with the receptor-ligand shape complementarity. To address this limitation, in 1958 Koshland introduced an *induced-fit* model to account for the local conformational changes observed in the receptor binding site [13]. According to this second model, upon binding the ligand induces local conformational changes in the receptor active site enhancing the receptor-ligand fit. A third *conformational selection* model – initially introduced by Pauling in 1940 [14] and subsequently adapted by Burgen and others [15-19] – gained popularity in the 1980s as a consequence of increasing knowledge on protein dynamics and the theoretical interpretation that biomolecules exhibit and interconvert between multiple, low energy conformations. According to the conformational selection model, the unbound receptor visits with a finite probability, the conformational states observed in the bound ensemble. In other words, the unbound ensemble includes relevant conformations of the receptors that are also contained in the bound ensemble. Hence, ligands may bind to these rare, transient conformations and shift the distributions from unbound to bound ensembles. Nuclear Magnetic Resonance (NMR) experiments have more recently confirmed the validity of such conformational selection model in various systems [20-23].

Lock-and-key, induced-fit, and conformational selection models were initially proposed as fundamentally general and mutually exclusive. However, recent studies provide evidence that these models are useful largely on a case-by-case basis (i.e. none of them can explain all molecular recognition scenarios). For systems with low shape complementarity, either induced fit or conformational selection models taken alone may not explain all the kinetic properties involved during molecular recognition processes [24]. Therefore, in several cases recognition processes are best modeled by integrating an initial phase of conformational selection followed by residual induced fit. A particularly relevant example is the case of ubiquitin. Lange et al. studied the ubiquitin protein using residual dipolar couplings (RDCs) in NMR experiment and showed the presence of conformational selection based on the structural similarity between the unbound ensemble measured by NMR and bound X-ray structures [22]. However, a rigorous theoretical analysis of the same experimental data by Wlodarski and Zagrovic focusing on the binding site conformational changes demonstrated the presence of residual induced-fit [25]. Peters and de Groot analyzed simulations of several ubiquitin complexes and recently proposed additional possible recognition models that go beyond induced fit and conformational selection typically considered [26].

Most of the studies investigating the mechanistic models of molecular binding rely on either local or global properties. Local properties are typically descriptors of the re-orientation of specific binding site residues upon binding [27]; global properties can be addressed by receptor structural similarity, for example, in terms of principal components (PC) of the atomistic fluctuations [22,28,29], or monitoring a distance between key distant functional groups [23]. However, quantifying the relative importance of induced-fit and conformational selection mechanisms is likely at variance with the specific properties of the system considered. Investigating these mechanistic models is an essential step for the design of LSD1 inhibitors and molecular probes targeting the H3-histone binding region.

In this study, we investigate the above-mentioned mechanistic models in the case of LSD1/CoREST-H3-histone molecular recognition using unbound and H3-histone bound conformational ensembles obtained from explicit solvent MD simulation. We undertake an extensive analysis of both local and global properties of LSD1/CoREST conformational changes using alternate metrics, including a previously proposed combined-clustering analysis [30]. A lock-and-key model can be immediately ruled out after inspection of the side chain conformational changes occurring upon binding in the H3-histone binding site. Instead, we find that the local conformational changes are compatible with the induced-fit model. We also show that conformational selection – despite in principle not ruled out by this finding – is minimal in this case and only relevant when global properties are considered, such as the nanoscale motion of the LSD1/CoREST clamp. While data from a total cumulative simulation of one microsecond was employed in this study, finite sampling artifacts may hide additional relevant dynamics of the LSD1/CoREST system. Future studies in our group will address this concern on the basis of focused, enhanced sampling methods for large amplitude motions.

Overall, our approach underscores the importance of addressing alternative binding mechanisms using multiple metrics. Our study provides a starting point for the future discovery of inhibitors and molecular probes targeting LSD1/CoREST H3-histone binding site, while including protein dynamics determinants of substrate recognition and binding.

## Results and discussion

Different analyses were employed to address the hypothesis whether conformational selection and/or induced-fit occur upon H3-histone binding to the LSD1/CoREST.

**Figure 2:**
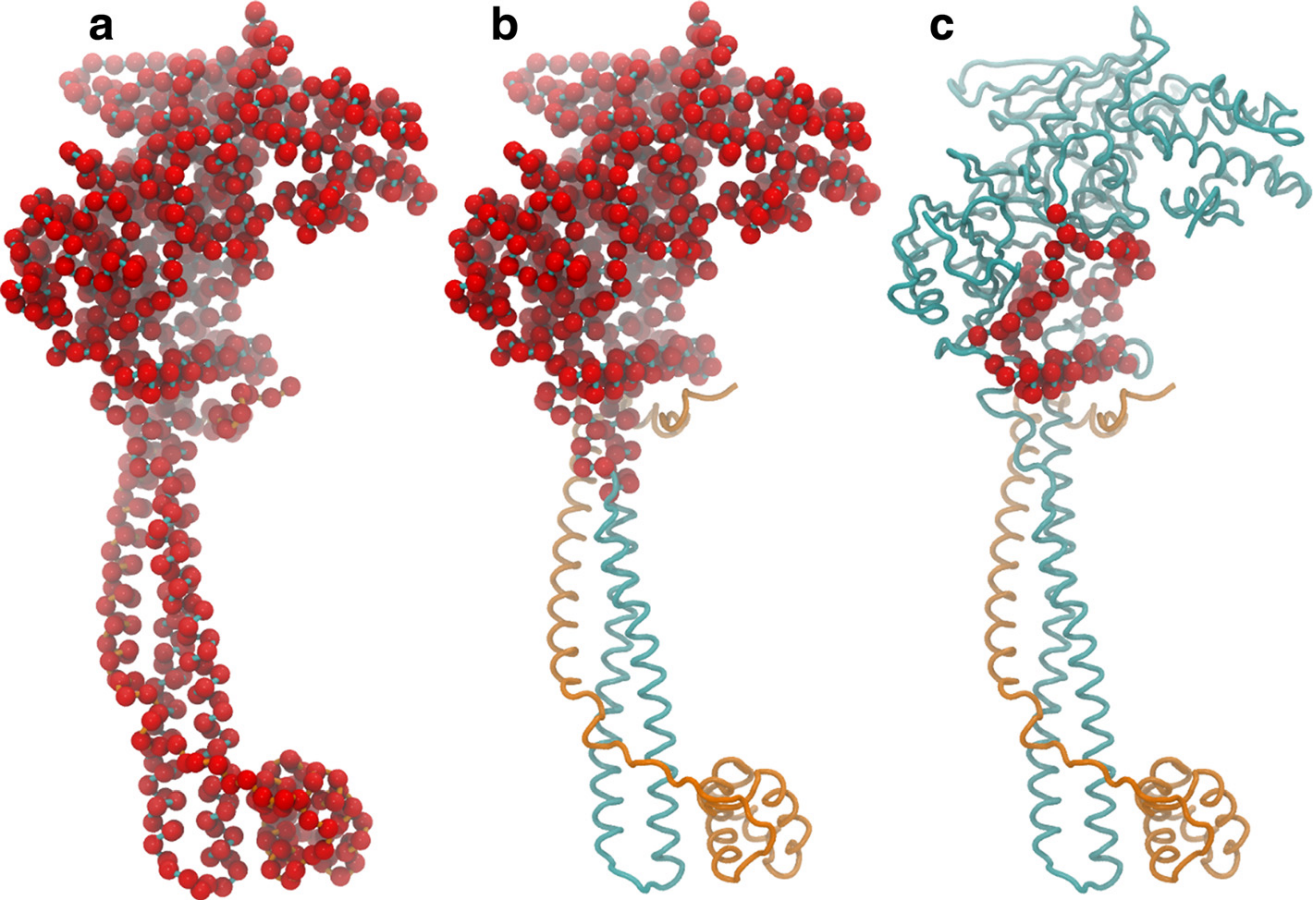
**Selection schemes used for analysis of the LSD1/CoREST-H3-histone binding mechanism. (a)** The whole LSD1/CoREST complex; **(b)** the truncated AO domain; and **(c)** the H3-histone binding site region. C^α^ atoms only were considered in each case wherever else specified are highlighted by red spheres. See also Figure 1 for a representation of LSD1/CoREST structural biology.

Based on the observation of conformational changes in the H3 histone binding regions, lock-and-key model was immediately ruled out to explain the molecular recognition. The induced-fit or conformational selection models were further investigated based on alternative analysis and metrics using three atomic selection schemes, as summarized in Figure 2: the whole system, the AO domain only, and the H3-binding site only. See Methods section for a detailed description of the computational analysis.

### Principal component analysis

**Figure 3:**
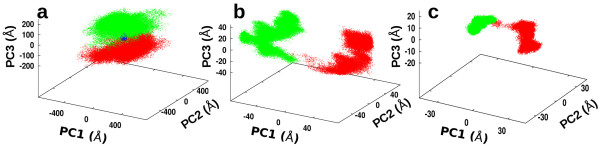
**Principal component analysis of the LSD1/CoREST atom fluctuations depending on the region of the LSD1/CoREST.** The unbound (red) and H3-bound (green) conformational ensembles were analyzed for **(a)** all C^α^-atoms in LSD1/CoREST, **(b)** the C^α^-atoms in the AO domain only, and **(c)** all atoms of the H3-binding site residues **(c)**. Data points in regions that overlap are highlighted in blue. See also Figure 2 for a structural identification of the three regions considered.

To investigate globular motions in the combined unbound and H3-bound conformational ensembles, principal component (PC) analysis was performed. Figure 3 displays the PC spaces for all combinations of first three most dominant PCs, which account alone for 86% (whole system), 84% (AO domain), and 87% (binding site) of the total atomic fluctuations for each region (see also Additional file 1: Table S1 in supporting information). Overlap of unbound and H3-bound ensembles in the PC space indicates exploration of similar conformations, thus reveals the presence of conformational selection upon binding.

The PC analysis of the unbound and H3 bound conformations clearly shows that overlap in PC space is minimal or absent and at variance with the structural region of LSD1/CoREST considered for the analysis. For example, using the ‘whole’ LSD1/CoREST selection scheme some minor overlap between unbound and H3-bound ensembles can be observed in the PC space (Figure 3a) in line with a conformational selection model. However, the data points that fall in these overlapping regions are only < 5% of the total. Moreover, a detailed structural analysis shows that such small overlap is due to the large molecular fluctuation of the LSD1/CoREST clamp captured in the PC space when the ‘whole’ selection scheme was considered [11]. Instead, when the ‘AO domain’ or ‘H3-binding site’ was considered as selection schemes, no overlap is found, ruling out conformational selection as preferred recognition model (see Figure 3b and c). We note that analysis based on the first most relevant PC is well motivated in this case, as the contribution of additional components is very minimal (at maximum 5% for all regions considered; see Additional file 1: Table S1). This assumption was also validated using all unique pair combination of the first five PC, and similar conclusions can be drawn for all selection schemes considered (not shown). Overall, our data is consistent with an induced-fit mechanism for LSD1/CoREST-H3-histone association.

### Kolmogorov-smirnov tests

**Figure 4:**
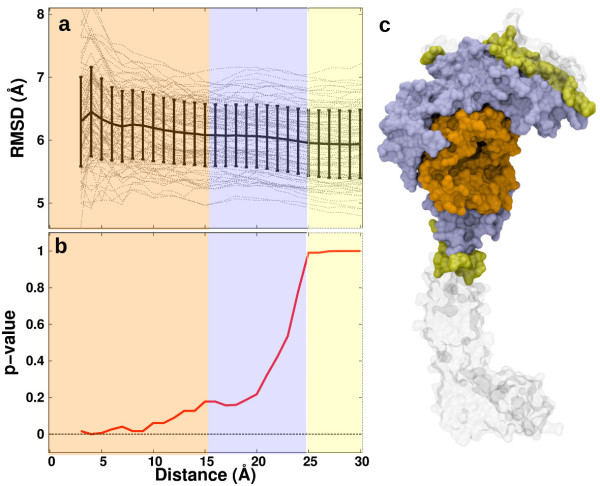
**Statistical relevance of induced-fit vs. conformational selection mechanisms as a function of increasing distance from the H3-histone site examined by Kolmogorov-Smirnov tests. (a)** The pairwise root-mean-square-deviation (RMSD) values between unbound and H3-bound conformation as a function of cumulative incremental distances of 1-Å from the binding site center of mass (gray lines); the average RMSD values (black line) and their standard deviation (vertical bars) are also shown. **(b)** p-values obtained using Kolmogorov-Smirnov tests (red line), comparing the statistical relevance of induced-fit vs. conformational selection as a function of the radial distance from the H3-histone site center of geometry; the background color coding corresponds to panel **(c)**. **(c)** Structural regions of the LSD1/CoREST nanoscale clamp highlighted based on the p-value ranges. Note that the inner region encompasses entirely the H3-binding site. See also the Methods section for analysis details.

We further analyzed the weak conformational selection cases emerging from the PC-based analysis described in the previous section when ‘whole’ system was used as selection scheme. To this end, the root mean square deviations (RMSD) between all unique pairs of unbound and H3-bound conformations were calculated as a function of distance from the center of mass of the H3-binding site up to a shell of 30 Å, which encompasses the entire AO domain (Figure 4a and c). The average RMSD values and their standard deviation values are slightly higher closer to the H3-binding site compared to the RMSD distribution at increasing distances. Are local and global RMSD data distributions statistically different?

This question can be addressed using a two-sample Kolmogorov-Smirnov (KS) p-value analysis. The average RMSD values as a function of distance from the H3-binding site were compared with the reference global deviations at a distance of 30 Å by means of p-values in standard KS tests (Figure 4b and c). The profile of p-values as a function of distance shows the statistical significance of the assumption of an induced-fit model vs. a conformational selection model. For example, a p-value of 0 signifies that the local RMSD distribution is statistically different compared with the global RMSD, thus demonstrating the statistical relevance of an induced-fit mechanism. On the other hand, a p-value of 1 implies no significant difference in local vs. global RMSD distribution; such a scenario in which local deviations are similar to global cannot be used to qualitatively justify either induced fit mechanism or conformational selection as the preferred mechanism. We find that p-values are zero up to a distance of 10 Å and increases in the range 10–25 Å. This 10 Å region encompasses entirely the H3-binding site. At distances beyond 25 Å, p-values are constantly one. This statistical analysis highlights that the molecular region surrounding the binding site of H3-histone undergoes conformational changes upon binding that are on average significantly different compared to the rest of the system. Overall, our KS-analysis data clearly shows that an induced fit model is representative as a molecular binding mechanism.

### Combined conformational clustering analysis

We tested the hypothesis whether conformational selection and/or induced-fit models are relevant by using an additional, alternative approach. The presence of H3-bound LSD1/CoREST receptor structures in the unbound ensemble or *vice-versa* of unbound structures in the H3-bound ensemble can be systematically verified using an RMSD-based combined-clustering analysis [30,31] in which both ensembles are simultaneously probed using a structure similarity criterion. Sensitivity of the analysis was evaluated on two input parameters, namely the RMSD similarity threshold values, and the atomic selection scheme. When similarity is evaluated over a threshold range physically relevant, presence of conformations from both ensembles within a same cluster suggests the presence of conformational selection. On the contrary, clusters populated by structures from either the unbound or the H3-bound ensemble indicates that these ensembles are distinctly different, in line with an induced-fit mechanism for molecular recognition.

**Figure 5:**
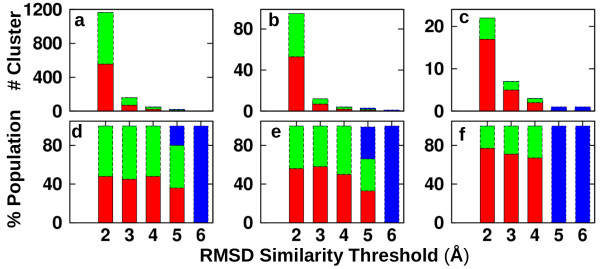
**Analysis of conformational selection based on a combined conformational clustering approach.** Number of clusters obtained as a function of RMSD similarity threshold used for clustering analysis for different selection schemes (**a**: ‘whole’; **b**: ‘AO domain’; **c**: ‘H3-binding site’) and corresponding relative populations (**d**: ‘whole’; **e**: ‘AO domain’; **f**: ‘H3-binding site’). Red: clusters with members only from the unbound MD ensemble. Green: clusters with members only from the H3-bound MD ensemble. Blue: clusters with members from both unbound and H3-bound MD ensembles. Conformational clustering was performed using the selection schemes in Figure 2.

Figure 5a shows the number of clusters obtained as a function of the RMSD similarity threshold used to distinguish structures. Using the ‘whole’ selection scheme, 1165 clusters are obtained with a threshold of 2 Å. The number of clusters decreases as a function of the RMSD threshold; namely, 158 clusters with a 3 Å threshold; 48 clusters with a 4 Å threshold; 22 clusters with a 5 Å threshold. This trend continues till the extreme scenario found when the RMSD threshold is increased to 6 Å in which all structures in the combined ensemble fall in 1 single cluster. As expected, increasing the RMSD threshold beyond 5 Å is unnecessary, as both unbound and H3-bound ensemble become indistinguishable. On the other hand, RMSD thresholds lower than 2 Å generate a significant number of singleton clusters (e.g. a threshold of 1 Å generated, 35,225 clusters, 26% of which populated by singleton clusters). Figure 5b and 5c summarize the results from similar analyses for the ‘AO domain’ and ‘H3-binding site’ selection schemes, respectively. Identical trends are observed in the three selection scenarios. Hence, clustering analysis was focused in the range of 2–5 Å RMSD threshold values. The corresponding relative cluster populations are shown in Figure 5d-f.

Our data indicates that in order to support a conformational selection scenario, i.e. MD snapshots common to the unbound and H3-bound ensemble, one has to increase the RMSD similarity threshold values beyond 4 Å (Figure 5). However, such a similarity criterion is in fact too weak for the three-selection scheme considered (structure pairs within such RMS deviation are not similar, indicating an artifact). Remarkably, the conformational clustering analysis presented overall supports the observation that LSD1/COREST recognition of the H3-histone tail is accompanied by significant alteration of the binding following an induced-fit model.

### LSD1 induced-fit upon H3-histone binding: simulation vs. experiment

While the clustering analysis uses RMSD similarity as a criterion to analyze all unique pairs of structures from different MD conformational ensembles, one can also use RMSD similarity as a metric to compare MD-generated ensembles with an experimental X-ray model. The X-ray models thus far reported for LSD1/CoREST bound to substrates have significantly similar binding site conformations. For example, PDB ID 2V1D [10] (bound with the H3-histone N-terminal peptide) and PDB ID 2Y48 [32] (bound with the SNAIL1 transcription factor N-terminal peptide) show RMSD values of 0.46 or 0.75 Å when the backbone or the ‘H3 binding site’ heavy-atoms were used, respectively.

**Figure 6:**
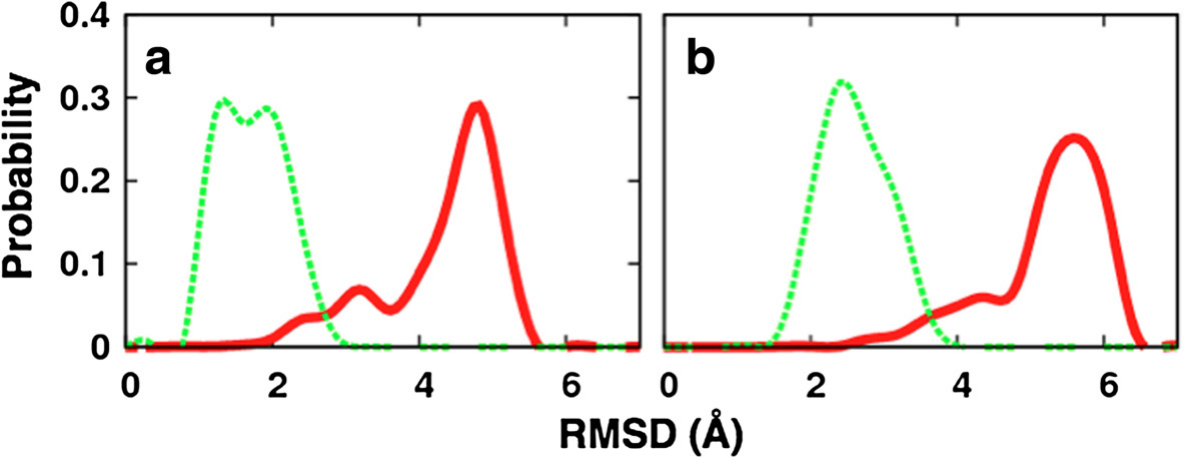
**Distributions of the root-mean-square deviation of MD snapshots from the reference X-ray structure (PDB ID: 2V1D).** Red: unbound MD ensemble. Green: H3-bound MD ensemble. Either **(a)** backbone or **(b)** all-heavy atoms of the residues lining the H3-binding site were considered for least-square fitting and RMSD calculation.

**Figure 7:**
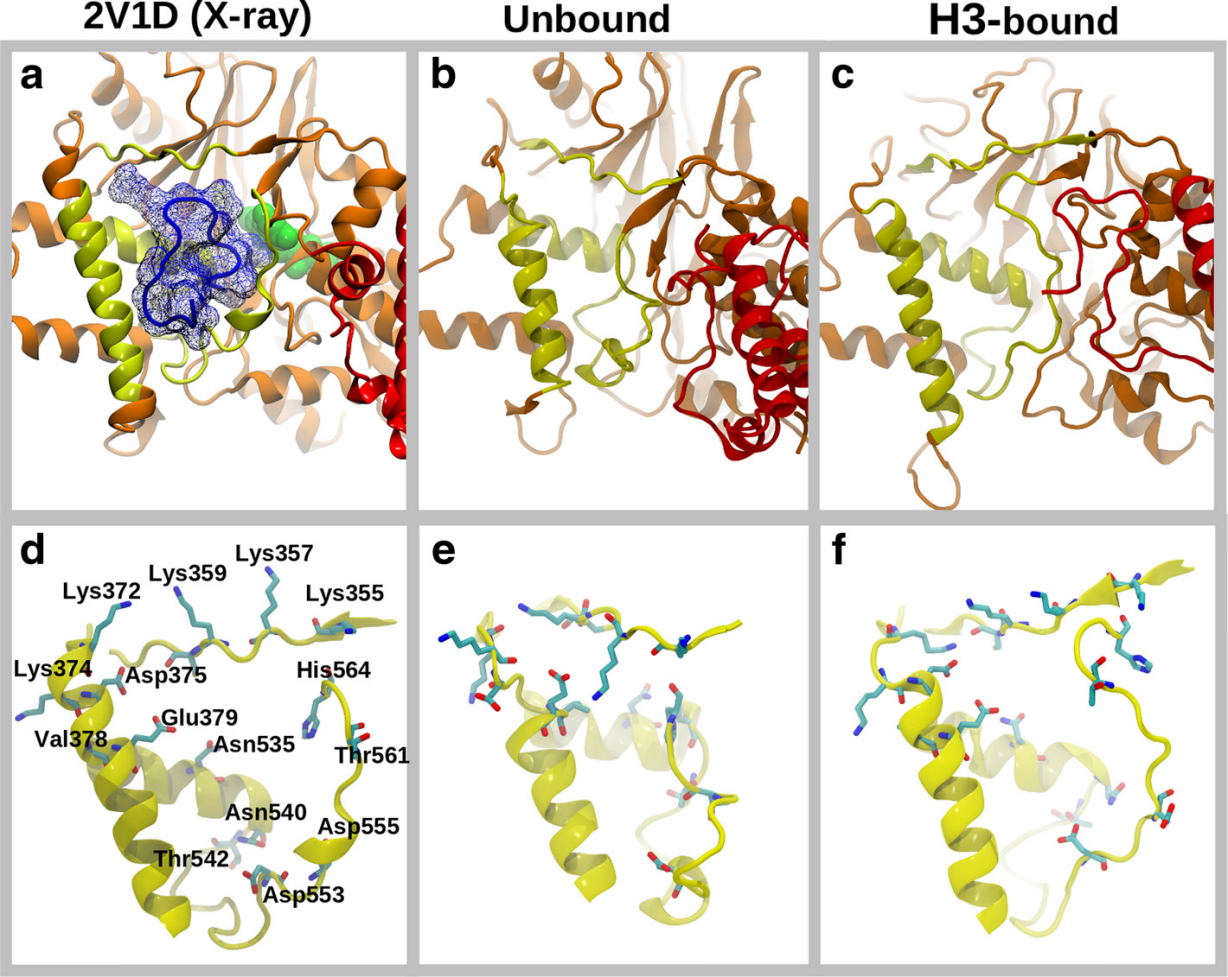
**LSD1/CoREST local conformational changes upon H3-histone binding are consistent with an induced-fit model. (a)** Reference X-ray structure (PDB entry 2V1D). Representative conformations from molecular dynamics **(b)** unbound and **(c)** H3-bound conformational ensembles highlight the residues involved in the induced-fit mechanism, as detailed in panels **(d-f)**.

Figure 6 summarizes the conformational diversity in the ‘H3-binding site’ comparing the 2V1D X-ray model with the structures in the MD ensembles in terms of RMSD values. The average RMSD values are 1.9 Å (backbone) and 2.8 Å (heavy-atoms) for the H3-bound ensemble, and 4.3 Å (backbone) and 5.3 Å (all-heavy atoms) for the unbound ensemble. Expectedly, the H3-bound MD ensemble is significantly more similar to the H3-bound crystal structure compared with the unbound MD ensemble, in line with the nature of the induced-fit mechanism. Based on the root-mean-square fluctuation (RMSF) analysis (see Additional file 1: Figure S1 in supporting information), residues in the H3-binding region that adopt more heterogeneous conformations in the unbound states are Lys355, Lys357, Lys372, Lys374, Val378, Asn540, Thr542, Asp555, Thr561 as represented in Figure 7. We speculate that the plasticity of the H3-histone binding site could be possibly exploited by nature to recognize various binding partners, such as the SNAIL/Scratch superfamily [32]. This observed molecular flexibility of the H3-histone binding site will be included in future research to discover and design molecular probes and inhibitors.

## Conclusion

The Lysine Specific Demethylase-1 (LSD1) enzyme in complex with its co-repressor protein CoREST is an epigenetic target of outstanding interest for the discovery of drugs against cancer and neurodegenerative disorders. Here, we investigated alternative mechanisms for the molecular recognition of the H3-histone using various conformational analyses and metrics. Our simulation data indicate that LSD1/CoREST non-covalent binding of the H3-histone follows preferably an induced-fit model to reorganize the binding site significantly. Both the side-chains and the backbone atoms of the residues in the H3-histone binding site are involved in the induced-fit mechanism. As previously noted by Wlodarski and Zagrovic [25], conformational selection might be operative when certain global properties are considered and often is at variance at the metric used for analysis, with residual induced fit being relevant at the binding site. Overall, this study shows that testing hypothetical molecular binding mechanisms depends in part on the metric used and the LSD1/CoREST structural region selected for analysis. Therefore, in general alternative approaches should be preferably undertaken for a reliable understanding of the molecular mechanisms underlying receptor-ligand binding. This study will aid the ongoing discovery and design of LSD1 inhibitors and molecular probes to investigate the detailed mechanism of LSD1/CoREST-H3-histone molecular recognition.

## Methods

### Molecular dynamics simulations

The previously reported 500 ns MD simulations from unbound and H3-bound runs were employed for this study and define two independently generated conformational ensembles. The unbound simulation of the LSD1/CoREST complex was initialized based on the X-ray crystal structure by Yang et al. [33] (PDB entry 2IW5) and a corresponding H3-histone N terminal tail (16 residues) bound simulation was initialized using the PDB entry 2V1D [34]. In this study, the initial 10 ns of simulation were discarded for equilibration. Molecular simulations were performed using the GROMACS simulation program (version 4.5.5 compiled in double precision [35]) and the GROMOS 53a6 force field [36] using explicit SPC water model [37] and compatible ion parameters [38]. MD snapshots were extracted every 10 ps along the two 500 ns periods and used for analysis (i.e. 50,000 snapshots for each ensemble). The details of these simulations were presented elsewhere [11].

### Analysis of the models for molecular recognition

Conformational analyses were performed to investigate the mechanism of LSD1/CoREST and H3-histone non-covalent binding, namely PC analysis, conformational clustering analysis and Kolmogorov-Smirnov (KS) statistical analysis (to probe if residual induced fit after conformational selection is observed). In all cases, the analysis was repeated using three different C^α^ atom-selection schemes (Figure 2), in order to address each binding mechanisms depending on the structural region of LSD1/CoREST considered: (a) the whole LSD1/CoREST complex; (b) a truncated AO domain only (residues Pro171-Glu427 and Ser517-Leu836); and (c) the H3-histone binding site (residues Leu353-Tyr363, Val370-Tyr391, Leu529-His564). Unbound and H3-bound trajectory ensembles were separately analyzed; a combined trajectory in which these trajectories were concatenated was also used in some cases as specified in the following of this section.

#### (a) principal component analysis

A concatenated ensemble of bound and unbound conformations was used for principal component (PC) analysis and the bound and unbound ensembles were projected separately on the combined set of principal modes. PC analysis was performed using all C^α^ atoms on all three atom-selection scheme considered. However, in the case of “whole” as selection scheme, individual frames of the unbound and H3-bound trajectory were aligned to 2IW5 crystal structure using C^α^ atoms of the tower domain of the LSD1 (residues Ile428-Pro516). The tower domain behaves essentially as a rigid body throughout both simulations and for this reason is an ideal choice for structural superimposition [11]. PC analysis [39,40] of the aligned conformational ensembles was performed using the Bio3D software [41], and mapping specific configurations from the unbound and H3-bound ensembles onto the PC space.

#### (b) combined conformational clustering analysis

In order to systematically find the occurrences of unbound conformation in the bound simulation, thus validating a conformational selection model, a previously described combined clustering approach was also employed [30]. This approach clusters MD snapshots from a combined ensemble of the unbound and H3-bound simulations based on RMSD similarity threshold values. For the clustering analysis of the combined trajectory, only the C^α^ atoms were considered and the GROMACS analysis tool ‘*g_cluster*’ was used. The trajectory was clustered using a RMSD based algorithm [31], after superimposing the conformations from the ensemble by means of a least-square-fitting procedure [42]. All C^α^ atoms for each of the atom-selection scheme previously described were considered for structural superimposition and conformational clustering.

#### (c) Kolmogorov-Smirnov statistical analysis

In order to probe residual induced-fit in the H3-histone binding site, a two-sample Kolmogorov-Smirnov (KS) statistical analysis was performed [43,44], following recent work by Wlodarski and Zagrovic on ubiquitin [25]. The KS test is a well-known statistical significance test with a null hypothesis that the compared data distributions are drawn from the same continuous distribution. For a cumulative distribution, KS statistics reads:

$$D_{\mathrm{nn}\prime }=\sup_{x}\left|F_{n}\left(x\right)-F_{n\prime }\left(\left(x\right)\right|\right)$$

where, *F*_*n*_(*x*) and *F*_*n* ′_(*x*)| are the two compared empirical distribution functions for the first and second data sets, respectively. If the data are from a same distribution, *D*_*nn* ′_ converges to zero. The null hypothesis is rejected on an α-level of significance when

$$\sqrt{\frac{\mathrm{nn}\prime }{n+n\prime }}D_{\mathrm{nn}\prime }>K\left(a\right),$$

where Κ(α) is obtained from

$$\mathrm{Pr}\left(K\leq K_{a}\right)=1-a,$$

and Κ is drawn from the Kolmogorov distribution.

Using PC space mapping of unbound and H3-bound conformation, for a given H3-bound conformation, the closest unbound conformation in the PC space was selected. Using this procedure, 94 such pairs were extracted using a cutoff distance of 50 units in PC space. The very fact that this selection of unbound and H3-bound by definition can be considered for a case for conformational selection in the PC space, in terms of global rmsd involving large conformational fluctuations. Using these pairs, RMSD between unbound and H3-bound conformations were calculated using the C^α^ atoms. These RMSD values were calculated as a function of distance from the center of mass of the H3-binding site at cumulative increments of 1 Å distances (the H3 peptide in the bound conformation was used to identify this arbitrary location). We note that the cumulative nature of these increments means that atom falling within a certain distance cutoff are also counted beyond that cutoff for higher distances. To differentiate if the magnitudes of local vs. global set of RMSD values are from the same RMSD distribution, KS analysis was performed. Each set of RMSD obtained as a function of distance was compared at a reference distance of 30 Å, and p-value was obtained from KS test for statistical comparison. In all cases, RMSD values were calculated after least square fitting [42] using the same subset of atoms used for the calculation of RMSD values.

### Availability of supporting data

Root-mean-square fluctuation plot for the binding site residues (Additional file 1: Figure S1) and percentage population of the PC rank (Additional file 1: Table S1), supporting the results of this article are included as additional files.

## Abbreviations

CoREST: Co-repressor RE1-Silecing Transcription factor protein; H3: Histone 3; MD: Molecular dynamics; LSD1: Lysine-Specific Demethylase-1; AO: Amine-oxidase; FAD: Flavin adenosine dinucleotide; RMSD: Root-Mean-Square Deviation; PCA: Principal component analysis; PDB: Protein data bank; KS: Kolmogorov Smirnov; NMR: Nuclear magnetic resonance.

## Competing interests

The authors declare that they have no competing interests.

## Authors’ contributions

NAV and RB designed the research; NAV performed the research; NAV and RB analyzed the data and discussed the results; and NAV and RB wrote the paper. Both authors read and approved the final manuscript.

## Supplementary Material

Additional file 1 **Figure S1. **Root-mean-square fluctuation (RMSF) of the H3-binding site. Only the C^α^ atoms of the H3-binding site were used for RMSF calculation. The RMSF for regions of the binding site are shaded as yellow, white and blue for residues 353–363, 370–391 and 529–564 respectively. **Table S1.** PCA rank for the three-selection scheme considered and corresponding relative contributions. Click here for file
