# Supplementary material for: Molecular dynamics simulations indicate an induced-fit mechanism for LSD1/CoREST-H3-histone molecular recognition
Source: BMC Biophys. 2013 Nov 25;6:15. doi: 10.1186/2046-1682-6-15 (PMC4175114; doi:10.1186/2046-1682-6-15)
Supplement: Additional file 1 — Figure S1. Root-mean-square fluctuation (RMSF) of the H3-binding site. Only the Cα atoms of the H3-binding site were used for RMSF calculation. The RMSF for regions of the binding site are shaded as yellow, white and blue for residues 353–363, 370–391 and 529–564 respectively. Table S1. PCA rank for the three-selection scheme considered and corresponding relative contributions. [file 2046-1682-6-15-S1.pdf]

Supporting Information

Molecular Dynamics Simulations Indicate an Induced-Fit Mechanism for LSD1/CoREST-H3-Histone Molecular Recognition

Nadeem A. Vellore and Riccardo Baron*

Department of Medicinal Chemistry, College of Pharmacy, and The Henry Eyring Center for Theoretical Chemistry, The University of Utah, Salt Lake City, UT 84112-5820, U.S.A.

*Corresponding author: Phone: +1-801-585-7117; Email: r.baron@utah.edu

Figure-S1: Root-mean-square fluctuation (RMSF) of the H3-binding site. Only the C^{α} atoms of the H3-binding site were used for RMSF calculation. The RMSF for regions of the binding site are shaded as yellow, white and blue for residues 353-363, 370-391 and 529-564 respectively.

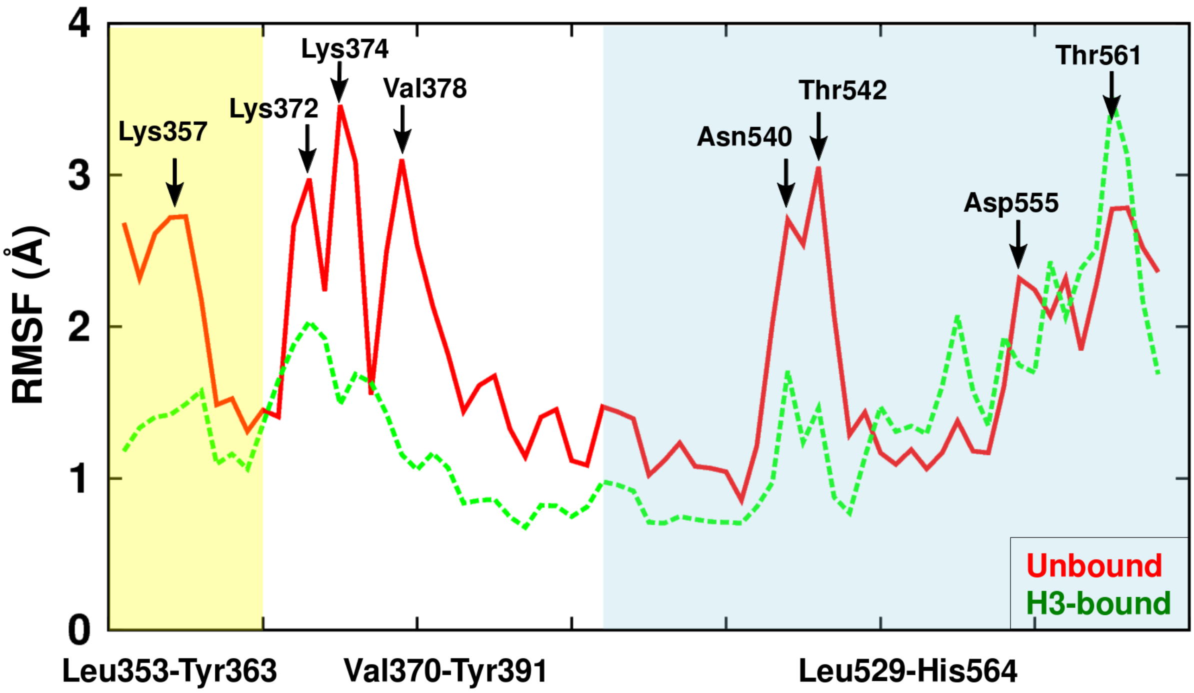

Table-S1: PCA rank for the three-selection scheme considered and corresponding relative contributions.

PC rank	Whole	AO domain	H3-binding site
	Percentage population (cumulative)		
1	37 (37)	55 (55)	71 (71)
2	31 (67)	16 (71)	9 (80)
3	19 (86)	13 (84)	7 (87)
4	5 (91)	5 (89)	3 (90)
5	4 (95)	4 (93)	2 (92)
